# [Corrigendum] Influence of MLH1 on colon cancer sensitivity to poly(ADP-ribose) polymerase inhibitor combined with irinotecan

**DOI:** 10.3892/ijo.2026.5868

**Published:** 2026-03-09

**Authors:** Lucio Tentori, Carlo Leonetti, Alessia Muzi, Annalisa Susanna Dorio, Manuela Porru, Susanna Dolci, Federica Campolo, Patrizia Vernole, Pedro Miguel Lacal, Françoise Praz, Grazia Graziani

Int J Oncol 43: 210-218, 2013; DOI: 10.3892/ijo.2013.1932

Following the publication of the above article, an interested reader drew to the attention of the Editorial Office that the immunofluorescence images shown in Fig. 3B on p. 214 to represent the 'HCT116 SiP' and 'HCT116+3 SiP' experiments (top row of data) were strikingly similar.

Upon examining their data, the authors have realized that the data in Fig. 3B were presented incorrectly; specifically, the data shown in the 'HCT116+3 SiP' panel were erroneously duplicated from the data shown correctly in the 'HCT116 SiP' panel.

The authors have now submitted a revised version of Fig. 3, containing the corrected version of the 'HCT116+3 SiP' data panel in Fig. 3B, and this is shown on the next page. Note that this error did not affect the overall conclusions reported in the study. The authors are grateful to the Editor of *International Journal of Oncology* for allowing them this opportunity to publish a Corrigendum, and all the authors agree with its publication. Furthermore, the authors apologize to the readership for any inconvenience caused.

## Figures and Tables

**Figure 3:**
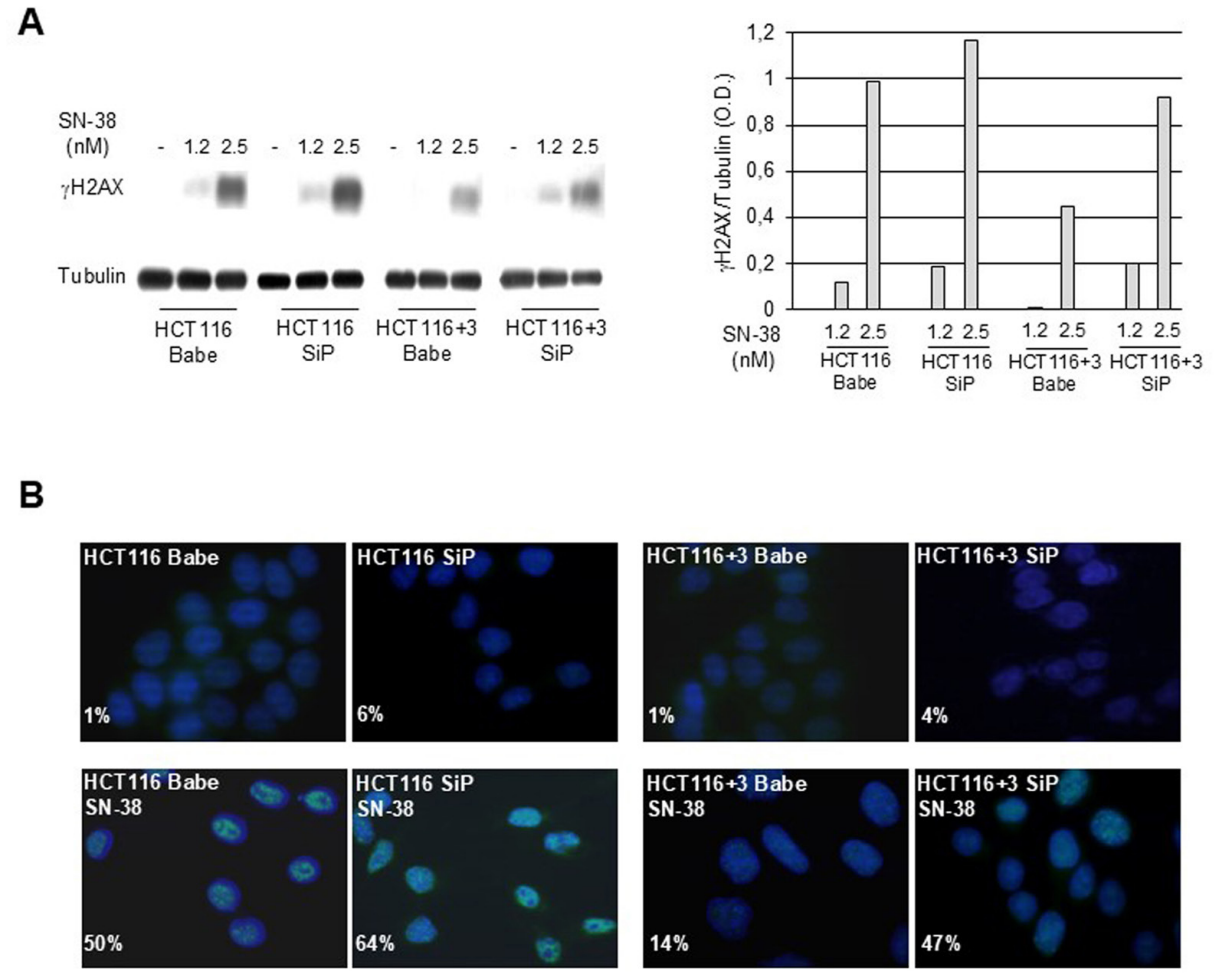
Analysis of DNA damage induced by SN-38 in HCT116 or HCT116+3 cells control or stably silenced for PARP-1 expression. (A) Immunoblot analysis of γ-H2AX expression in SN-38 treated cells. Cells were treated with SN-38 (1.2 and 2.5 nM) for 24 h and analysed for the expression of γ-H2AX or tubulin. Histograms represent the ratios between the OD of γ-H2AX in SN-38 treated groups and tubulin. The results are representative of one out of two experiments with similar results. (B) Immunofluorescence analysis of γ-H2AX foci (green) in untreated or SN-38 treated cells. Nuclei were stained with DAPI (blue). The percentage of cells with ≥5 γ-H2AX foci of one representative out of two experiments is presented (50 cells counted for each experiment).

